# Analysis of Predictive Equations for Estimating Resting Energy Expenditure in a Large Cohort of Morbidly Obese Patients

**DOI:** 10.3389/fendo.2018.00367

**Published:** 2018-07-25

**Authors:** Raffaella Cancello, Davide Soranna, Amelia Brunani, Massimo Scacchi, Antonella Tagliaferri, Stefania Mai, Paolo Marzullo, Antonella Zambon, Cecilia Invitti

**Affiliations:** ^1^Obesity Research Laboratory, IRCCS Istituto Auxologico Italiano, Milan, Italy; ^2^IRCCS Istituto Auxologico Italiano, Milan, Italy; ^3^Division of Rehabilitation Medicine, IRCCS Istituto Auxologico Italiano, Piancavallo-Oggebbio, Italy; ^4^Division of Endocrinology and Metabolic Diseases, IRCCS Istituto Auxologico Italiano, Piancavallo-Oggebbio, Italy; ^5^Department of Clinical Sciences and Community Health, University of Milan, Milan, Italy; ^6^Laboratory of Metabolic Research, IRCCS Istituto Auxologico Italiano, Piancavallo-Oggebbio, Italy; ^7^Department of Translational Medicine, University of Piemonte Orientale, Novara, Italy; ^8^Department of Statistics and Quantitative Methods, Biostatistics, Epidemiology and Public Health, Milano-Bicocca University, Milan, Italy

**Keywords:** resting energy expenditure, indirect calorimetry, comorbidities, REE predictive equations, obesity

## Abstract

The treatment of obesity requires creating an energy deficit through caloric restriction and physical activity. Energy needs are estimated assessing the resting energy expenditure (REE) that in the clinical practice is estimated using predictive equations. In the present cross sectional study, we compared, in a large cohort of morbidly obese patients, the accuracy of REE predictive equations recommended by current obesity guidelines [Harris-Benedict, WHO/FAO/ONU and Mifflin-St Jeor (MJ)] and/or developed for obese patients (Muller, Muller BC, Lazzer, Lazzer BC), focusing on the effect of comorbidities on the accuracy of the equations. Data on REE measured by indirect calorimetry and body composition were collected in 4,247 obese patients (69% women, mean age 48 ± 19 years, mean BMI 44 ± 7 Kg/m^2^) admitted to the Istituto Auxologico Italiano from 1999 to 2014. The performance of the equations was assessed in the whole cohort, in 4 groups with 0, 1, 2, or ≥ 3 comorbidities and in a subgroup of 1,598 patients with 1 comorbidity (47.1% hypertension, 16.7% psychiatric disorders, 13.3% binge eating disorders, 6.1% endocrine disorders, 6.4% type 2 diabetes, 3.5% sleep apnoea, 3.1% dyslipidemia, 2.5% coronary disease). In the whole cohort of obese patients, as well as in each stratum of comorbidity number, the MJ equation had the highest performance for agreement measures and bias. The MJ equation had the best performance in obese patients with ≥3 comorbidities (accuracy of 61.1%, bias of −89.87) and in patients with type 2 diabetes and sleep apnoea (accuracy/bias 69%/−19.17 and 66%/−21.67 respectively), who also have the highest levels of measured REE. In conclusion, MJ equation should be preferred to other equations to estimate the energy needs of Caucasian morbidly obese patients when measurement of the REE cannot be performed. As even MJ equation does not precisely predict REE, it should be better to plan the diet intervention by measuring rather than estimating REE. Future studies focusing on the clinical differences that determine the high inter-individual variability of the precision of the REE predictive equations (e.g., on the organ-tissue metabolic rate), could help to develop predictive equations with a better performance.

## Introduction

The treatment of obesity requires creating an energy deficit through caloric restriction, physical activity, or both (1). The energy needs are based on the resting energy expenditure (REE) which is the major component of the total daily energy expenditure and reflects the energy required to maintain the vital functions at resting state. The major determinant of the REE is the fat- free mass (FFM), which is composed by the sum of two moieties, one with a high metabolic rate (skeletal muscle and visceral organs accounts for 16 and 84% of REE respectively) and one with a low metabolic rate (bone and extracellular mass) (2).

Early studies suggested that obesity is due to a predisposition to a lower REE that contributes with sedentary lifestyles to a positive energy balance (3). Conversely, subsequent studies demonstrated that REE levels are high in non-sarcopenic obese individuals because the body weight increase is associated with a concomitant increase in fat mass (FM) and FFM, and the FM also is an independent predictor of REE with greater effect in subjects with higher FM amount (3). Although it has not been established with certainty what the FFM compartment is the main predictor of REE in obese subjects, some studies support a greater role for the visceral component than muscular mass (2). The REE is commonly measured with indirect calorimetry (4), which however it is not used in the majority of outpatients because it is expensive and time consuming. Thus, in the clinical practice energy needs are estimated using REE predictive equations based on body weight, height, age and sex and/or body composition parameters. Several predictive equations have been proposed in the last century, however current guidelines for the management of obesity still recommend equations developed more than 30 years ago (Harris Benedict 1984, the FAO/WHO/ONU 1985) on populations definitely different from the nutritional point of view from the current ones (5, 6). Since the evidence that the application of Harris Benedict 1984 and FAO/WHO/ONU 1985 for determining REE in overweight/obese subjects tends to overestimate the true metabolic rate, the Academy of Nutrition and Dietetics recommends the use of the Mifflin-St Jeor equation in obese individuals (7–9). In a large cohort of obese subjects free of metabolic or endocrine diseases such as diabetes, hypertension, and hypothyroidism, Lazzer et al developed predictive equations based on age, sex and FFM finding that the measured REE was correctly predicted in 56% of adult subjects. The authors also reported that the accuracy of the predictive equation was not improved by the inclusion of FM in the formula (10).

A very recent external validation of REE predictive equations reported that the accuracy of the formulas decreases going from normal-weight to class 3 obesity (11). This suggests that in morbid obesity, there are factors affecting the REE that are not captured by the available equations. Considering that the probability of having multiple comorbidities increases with the degree of obesity, it could be hypothesized that one of the factors that affects the predictive capacity of the equations is linked to the altered metabolic rate of organs compromised by specific pathologies. In this regard, the REE is altered in patients with cardio-metabolic diseases such as type 2 diabetes, hypertension and sleep apnoea (12–14). For this reason, Huang et al. included a correction factor in a predictive equation specific for patients with type 2 diabetes (14).

In the present cross sectional study, we compared in a large cohort of morbidly obese patients, the accuracy of REE predictive equations recommended by current guidelines and/or developed for obese patients, focusing on the presence and type of comorbidity.

## Materials and methods

### Study design and participants

A cross sectional study was carried out on 4,247 adult Caucasian obese and morbidly obese patients admitted for a weight loss intervention from 1999 to 2014 at the IRCCS Istituto Auxologico Italiano, Piancavallo (Verbania, Italy). Patients were selected for being admitted for a weight loss intervention, being free of acute diseases and having a physiologic Respiratory Quotient (RQ between 0.71 and 1.0) during the indirect calorimetry measurement performed before the weight loss intervention. The following parameters were collected: age, sex, height, weight, FM, FFM, and REE assessed by indirect calorimetry, and the presence comorbidities (hypertension, type 2 diabetes, coronary disease, dyslipidemia, sleep apnoea, endocrine disorders psychiatric disorders and binge eating disorder). The IRCCS Istituto Auxologico Italiano Ethics Committee (https://www.auxologico.it/ricerca-formazione/comitato-etico, Via Ariosto 13, Milano, Italy, e-mail: comitato.etico@auxologico.it) approved the study and all subjects involved were informed and gave their signed consent to use data for research purposes.

### Indirect calorimetry and body composition

The REE was measured in the morning between 8 and 9 a.m. after a fasting period of 12 h in thermoneutral conditions (in a 22–25°C room) by an open-circuit, indirect computerized calorimetry (Vmax 29, Sensor Medics, Yorba Linda, CA, USA) which is periodically subjected to quality controls to ensure the reliability of the measures. The flow sensor calibration was completed daily after at least 30 min of warm up of the Vmax calorimeter prior to measurement session. On each testing day, the calorimeter performed two calibration points using two reference gas mixtures (15% O_2_/5%CO_2_ and 26% O_2_/0%CO_2_) and the calibration of the environmental gases, allowing measuring the physiological range of the inspired and exhaled volumes. Subjects were physically inactive for at least 12 h and not smoking from at least 8 h. Subjects were awake and in supine position with the head placed in a rigid, transparent ventilated canopy. The respiratory exchange was measured for 30 min or until the steady state (defined as no variations higher than 5% during 5 consecutive min) had been reached. Data of the 10 min acclimation period were discarded. Minute-by-minute measurements of CO_2_ (mL/min) expired and, O_2_ consumed (mL/min) and RQ (VCO_2_/VO_2_) were recorded. The REE was calculated using the Weir equation [Kcal/d = 1.44^*^(3.94VO_2_+1.11VCO_2_)].

The body composition was assessed by the bioelectrical impedance analysis (BIA 101 Anniversary, Akern, Florence Italy), in the morning after an overnight fast and no more than 2 days later the execution of the indirect calorimetry.

### REE predictive equations

The REE predictive equations used in this study are reported in Table 1. We choose the equations recommended for overweight and obese subjects by national and international guidelines and/or developed in large cohorts of obese individuals (5–10, 14–16).

**Table 1:** REE predictive equations used in the present study.

Harris Benedict 1984 (kcal/day)	*Male* 88.362 + 4.799*height (cm) + 13.397*weight (kg) − 5.677*age *Female* 447.593 + 3.098*height (cm) +9.247*weight (kg) − 4.330*age
Huang (kcal/day)	71.767 − 2.337*age + 257.293*sex (M = 1; F = 0) + 9.996*weight (Kg) + 4.132*height (cm) + 145.950*DM (non-diabetic = 0; diabetic = 1)
Mifflin-St Jeor (Kcal/day)	9.99*weight (Kg) + 6.25*height (cm) − 4.92*age + 166*sex (M = 1; F = 0) −161
Lazzer 2010 (kcal/day)	11*kg − 3*age + 272*sex (M = 1; F = 0) + 777
Lazzer BC 2010 (Kcal/day)	20*FFM (kg) − 2*age − 11*sex (F = 0; M = 1) + 841
Muller (BMI ≥ 30) (MJoule/day)	0.05*weight (kg) + 1.103*sex (F = 0; M = 1) − 0.01586*age + 2.924
Muller BC (BMI ≥ 30) (MJoule/day)	0.05685*FFM (kg) + 0.04022*FM (kg) + 0.808*sex − 0.01402*age + 2.818
FAO/WHO/ONU (Kcal/day)	*Male* 18–30 yrs 15.4*weight (Kg)−27*height (m) + 717 30–60 yrs 11.3*weight (Kg) + 16*height (m) + 901 ≥60 yrs 8.8 *weight (Kg) + 1,128*height (m) − 1,071 *Female* 18–30 yrs 13.3*weight + 334*height (m) + 35 30–60 yrs 8.7*weight − 25*height (m) + 865 ≥60 yrs 9.2*weight + 637*height (m) − 302

### Statistical analyses

Sociodemographic and clinical continuous variables were expressed as means ± standard deviation (SD) and categorical data as frequencies and proportions. The agreement between measured REE (reference) and REE obtained by the predictive equations was analyzed both graphical visualizations (limits of agreement, LOA) in the Bland-Altman plot (17) and several indices (accuracy, bias, concordance correlation coefficient, CCC, and Mean squared Deviation, MSD) (18). In brief, for each predictive equation, the Bland-Altman plot compares the predicted with the measured REE. Specifically, the comparison consists to plot the differences between the predicted REE and the measured REE on y-axis and the reference on x-axis (19). The LOA reported in the Bland-Altman plot give the range of discordance between predicted and reference that, with a confidence of 1-α, comprises the true value of discordance. The accuracy is the proportion of patients in which the predicted REE falls in the range of acceptability (±10% of the measured REE).

The bias is the mean of the differences between predicted and measured REE; positive values suggest overestimates of the measurements obtained by the considered specific predictive equation and negative values suggest the opposite. The Concordance Correlation Coefficient (CCC), varying between 0 and 1, measures the concordance between the predicted and measured REE. Finally the Mean Squared Deviation (MSD) represents the expectation of the squared difference between predicted and measured REE, with low values suggesting good agreement. All analyses were performed in the entire sample and in specific subgroups defined by number and type of comorbidities. Chi square and trend tests were used to test the differences between subgroups. All analyses were performed using SAS version 9.4 software (SAS Institute, Cary, NC, USA).

## Results

The Table 2 describes the clinical characteristics of the cohort of 4,247 obese patients considered in the sample. Seventy eight percent of patients had a concomitant disease. Psychiatric diseases included personality disorders, anxiety and obsessive-compulsive and phobic disorders. Most of endocrine diseases (70%) were represented by hypothyroidism, on L-thyroxin treatment, or subclinical hypothyroidism.

**Table 2:** Sociodemographic and clinical characteristics of the whole sample of 4,247 obese patients.

**Variable**	
Age, years	47.66 ± 13.85
Male, *n (%)*	1,300 (31%)
Weight, kg	117.80 ± 24.03
Height, cm	162.79 ± 9.83
BMI, kg/m^2^	44.33 ± 7.46
**BMI classes, *n (%)*:**	
30–35	319 (8%)
35–40	868 (20%)
40–45	1,302 (31%)
≥45	1,758 (41%)
Fat free mass, kg	63.6 ± 18.44
Fat mass, kg	56.3 ± 18.17
Concomitant diseases, *n (%)*	3,242 (76)
Hypertension, *n (%)*	2,095 (49)
Type 2 diabetes, *n (%)*	939 (22)
Coronary disease, *n (%)*	260 (6)
Dyslipidemia, *n (%)*	638 (15)
Sleep apnoea, *n (%)*	576 (14)
Endocrine disorders, *n (%)*	262 (6)
Psychiatric disorders, *n (%)*	597 (14)
Binge eating disorder, *n (%)*	438 (10)
REE measured, kcal/day	1875.41 ± 430.16
FAO/WHO/ONU, kcal/day	1989.8 ± 402.75
Harris Benedict 1984, kcal/day	1986.61 ± 412.97
Huang, kcal/day	1921.63 ± 362.92
Lazzer 2010, kcal/day	2013.14 ± 348.87
Lazzer BC 2010, kcal/day	2015.08 ± 371.84
Mifflin-St Jeor, kcal/day	1849.61 ± 346.88
Muller, kcal/day	2006.7 ± 370.96
Muller BC, kcal/day	1979.3 ± 431.03

*Data are expressed as mean ± SD or percentage*.

**Figure 1:**
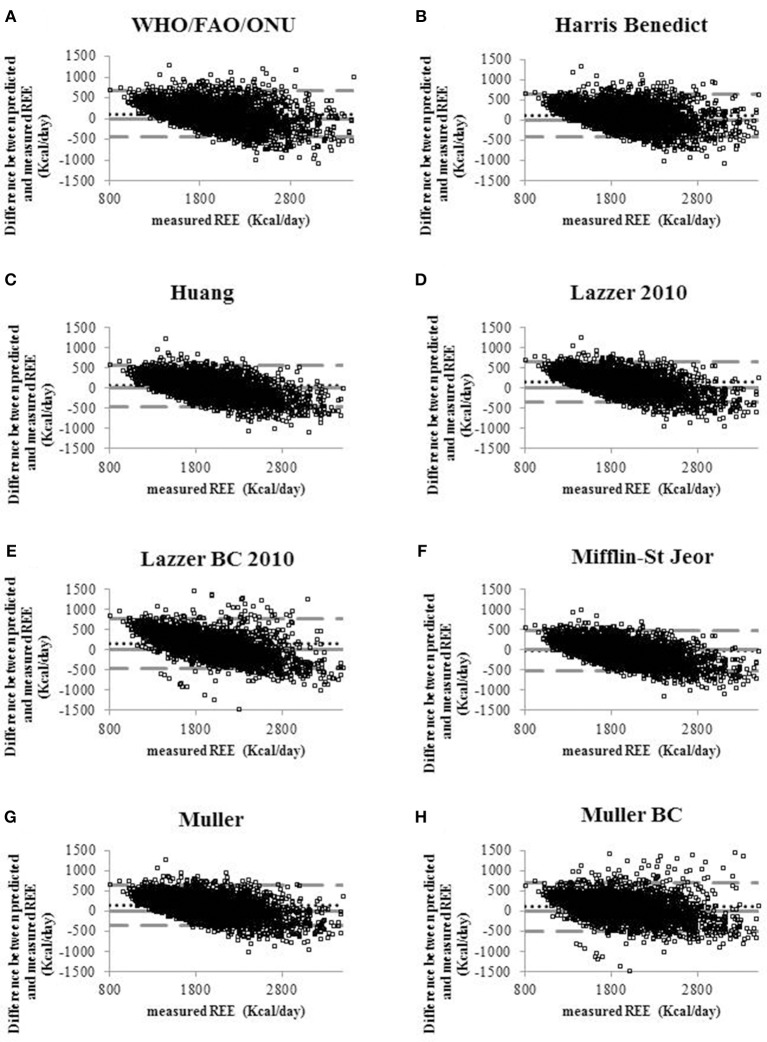
Bland-Altman plots displaying the agreement between measured REE and the REE predicted by eight predictive equations **(A)** WHO/FAO/ONU, **(B)** Harris Benedict 1984, **(C)** Huang, **(D)** Lazzer 2010, **(E)** Lazzer BC 2010, **(F)** Mifflin-St Jeor, **(G)** Muller, **(H)** Muller BC equations. Continuous lines indicate the value of the difference equal to 0 that means that the REE predicted coincides with REE measured. Dotted lines indicate the level of agreement of predicted and measured REE. Pointed lines indicate the mean of the differences between predicted and measured REE (bias).

Figure 1 shows the Bland-Altman plots for all predictive equations. The Mifflin-St Jeor equation had the lowest value of bias index which provides a mean measure of the overestimation or the underestimation of the specific equation compared to the measured REE (−25.80 kcal/day). These results fitted with the other agreement measures (accuracy, CCC and MSD), as reported in Table 3. The CCC of Mifflin-St Jeor equation was close to 80% with lowest value of MSD and the higher value of accuracy (56.18%). The accuracy of the Mifflin-St Jeor equation increases significantly in the tertiles of BMI (51.47, 57.93, and 58.08% in BMI < 40, 40–45.9, and >46 kg/m^2^ respectively, *p* < 0.001 for trend). Separation by sex and age tertiles did not affect the accuracy of Mifflin-St Jeor equation. When we stratified the sample by the number of comorbidities, 4 groups were considered (0, 1, 2, or ≥ 3 comorbidities). The proportion of patients of each group was 24, 38, 22, and 16%. Body mass index, measured REE and age (41.7 ± 13.7, 46.5 ± 13.9, 50.5 ± 12.6, and 55.3 ± 10.7 years, *p* < 0.001) increased from the group with 0 to the group with ≥3 comorbidities (Table 4). This finding was similar in the two sexes (data not shown). The accuracy of each predictive equation significantly improved with the increase in the number of comorbidities (*p* < 0.0001 for trend for each equation except for the Huang equation which was not significant). In each stratum, the Mifflin-St Jeor equation showed the higher performance for both agreement measures and bias (Table 5). In the more complicated obese patients, the Muller BC equation had a performance similar to the Mifflin-St Jeor equation (Table 5).

**Table 3:** Comparison of predicted with measured REE.

	**Accuracy (%)**	**Bias**		**CCC**	**MSD**
		**(P-M)**	%*		
FAO/WHO/ONU	47.12	114.39	6.1	0.75	89042.44
Harris Benedict 1984	49.28	111.20	5.9	0.78	79953.23
Huang	53.83	46.22	2.5	0.79	65021.93
Lazzer 2010	46.08	137.73	7.3	0.75	80354.97
Lazzer BC 2010	44.69	139.67	7.4	0.67	112628.42
Mifflin-St Jeor	56.18	−25.80	−1.4	0.79	63113.78
Muller	46.86	131.29	7.0	0.77	78981.30
Muller BC	49.12	103.89	5.5	0.74	97616.05

CCC, Concordance Correlation Coefficient; MSD, Mean Squared Deviation; BC, Body composition; P, REE predicted; M, REE measured; *Mean difference percentage calculated as the mean difference between REE predicted and REE measured divided by the mean of REE measured.

**Table 4:** Values of measured REE and BMI according to the number and type of comorbidity.

**Number of comorbidities**	***N* subjects**	**Measured REE (kcal.day^−1^)**	**BMI (kg/m^2^)**
0	1,005	1782.2 ± 405.09	42.1 ± 6.7
1	1,598	1845.7 ± 420.98	43.9 ± 7.7
2	955	1940.1 ± 434.55	45.9 ± 7.5
≥3	689	1990.7 ± 442.06	46.5 ± 6.9
*p*-value trend		< 0.0001	< 0.001
Type of comorbidity for patients with only 1 comorbidity			
Hypertension	754	1841.6 ± 427.26	44.41 ± 7.90
Psychiatric disorders	267	1791.6 ± 383.11	42.45 ± 7.77
Binge eating disorders	213	1821.1 ± 388.96	42.59 ± 6.88
Endocrine disorders	114	1681.0 ± 328.20	42.40 ± 6.65
Type 2 Diabetes	103	2059.4 ± 413.07	46.30 ± 5.91
Sleep Apnoea	56	2194.9 ± 469.10	49.20 ± 8.87
Dyslipidemia	50	1879.5 ± 453.43	42.71 ± 6.27
Coronary disease	41	1803.4 ± 418.85	41.32 ± 7.63

**Table 5:** Comparison of predicted with measured REE according to the number of comorbidity.

**Number of comorbidities**		**Accuracy (%)**	**Bias**		**CCC**	**MSD**
			**(P-M)**	%^*^		
0 (*N* = 1,005)	FAO/WHO/ONU	39.60	181.47	11.84	0.72	101766.30
	Harris Benedict 1984	43.28	154.77	10.34	0.75	85548.99
	Huang	51.74	49.07	4.58	0.79	59539.87
	Lazzer 2010	40.70	170.55	11.74	0.72	85803.17
	Lazzer BC 2010	36.12	202.58	13.82	0.62	134676.82
	Mifflin-St Jeor	51.74	28.22	3.34	0.79	59325.23
	Muller	41.69	163.38	11.10	0.73	84119.36
	Muller BC	41.19	143.42	9.36	0.71	111088.29
1 (*N* = 1,598)	FAO/WHO/ONU	44.49	125.90	8.75	0.72	95909.49
	Harris Benedict 1984	47.37	114.46	7.96	0.76	83854.69
	Huang	53.13	30.11	3.55	0.77	66744.29
	Lazzer 2010	43.99	143.20	10.03	0.73	85078.00
	Lazzer BC 2010	41.43	174.45	11.97	0.63	131743.88
	Mifflin-St Jeor	54.13	−12.12	1.20	0.77	65840.58
	Muller	44.49	136.66	9.45	0.74	83757.19
	Muller BC	44.81	135.80	8.91	0.71	113600.30
2 (*N* = 955)	FAO/WHO/ONU	52.98	78.37	5.45	0.79	77409.69
	Harris Benedict 1984	53.61	86.04	5.56	0.81	73905.39
	Huang	56.34	37.98	3.49	0.81	64472.79
	Lazzer 2010	50.58	116.40	7.79	0.79	72064.48
	Lazzer BC 2010	51.20	84.33	6.30	0.75	85019.39
	Mifflin-St Jeor	60.73	−59.30	−1.72	0.81	60849.02
	Muller	50.68	111.66	7.32	0.80	71655.95
	Muller BC	55.71	66.97	4.71	0.79	80940.12
≥3 (*N* = 689)	FAO/WHO/ONU	56.02	39.75	3.49	0.80	70990.02
	Harris Benedict 1984	56.46	74.98	4.96	0.82	71411.12
	Huang	55.01	90.85	6.26	0.79	70053.12
	Lazzer 2010	52.54	106.74	7.27	0.78	73206.67
	Lazzer BC 2010	55.73	43.98	4.30	0.76	74767.81
	Mifflin-St Jeor	61.10	−89.87	−3.15	0.80	65711.27
	Muller	54.57	99.22	6.67	0.80	70826.07
	Muller BC	61.54	23.37	2.85	0.80	64353.62

* *Mean difference percentage calculated as the mean difference between REE predicted and REE measured divided by the mean of REE measured. CCC, Concordance Correlation Coefficient; MSD, Mean Squared Deviation; BC, Body composition; P, REE predicted; M, REE measured*.

To verify whether the performance of the predictive equations was comorbidity-dependent, we measured the accuracy of the equations in 1,598 patients with only one comorbidity. Forty seven percent of them had hypertension, 16.7% psychiatric disorders, 13.3% binge eating disorders, 6.1% endocrine disorders, 6.4% type 2 diabetes, 3.5% sleep apnoea, 3.1% dyslipidaemia and 2.5% coronary disease (Table 4). The Mifflin-St Jeor equation had the highest performance values in patients with type 2 diabetes and sleep apnoea (accuracy/bias 69%/−19.17 and 66%/−21.67 respectively) (Table 6) who were also those with the highest levels of measured REE (Table 4).

**Table 6:** Accuracy (%) and Bias (kcal) of the REE predictive equations according to the single comorbidity in 1,598 patients with only one comorbidity.

	**Hypertension**		**Psychiatric disorders**		**Binge eating disorders**		**Endocrine disorders**		**Type 2 Diabetes**		**Sleep Apnoea**		**Dyslipidemia**		**Coronary disease**	
	**Accuracy**	**Bias**	**Accuracy**	**Bias**	**Accuracy**	**Bias**	**Accuracy**	**Bias**	**Accuracy**	**Bias**	**Accuracy**	**Bias**	**Accuracy**	**Bias**	**Accuracy**	**Bias**
FAO/WHO/ONU	48.01	87.39	39.33	141.50	39.91	180.75	38.60	177.60	49.51	156.89	42.86	191.28	46.00	137.35	41.46	122.67
Harris Benedict 1984	48.67	90.62	44.19	109.77	46.48	143.09	46.49	155.68	57.28	154.51	42.86	203.47	42.00	121.28	39.02	89.48
Huang	51.99	12.45	54.31	12.97	57.28	34.50	47.37	65.85	56.31	164.58	57.14	28.89	56.00	24.40	43.90	15.29
Lazzer 2010	43.63	133.85	39.70	136.02	44.13	156.96	43.86	192.41	56.31	143.45	48.21	161.71	42.00	143.14	43.90	127.94
Lazzer BC 2010	40.45	189.14	38.95	179.33	39.91	190.87	33.33	222.50	64.08	54.61	51.79	47.81	40.00	153.86	36.59	152.53
Mifflin-St Jeor	51.72	−43.03	54.31	16.57	57.28	39.78	50.00	55.14	68.93	−19.17	66.07	−21.67	52.00	−24.84	41.46	−41.03
Muller	45.09	121.51	37.83	133.86	43.66	157.98	44.74	183.90	59.22	149.52	46.43	175.71	42.00	133.32	43.90	109.77
Muller BC	43.77	150.07	42.32	135.32	44.60	143.24	37.72	151.33	37.72	151.33	48.21	75.63	50.00	112.68	39.02	68.14

The accuracy of Mifflin-St Jeor equation was significantly higher in diabetic than in non-diabetic patients (68.93 vs. 53.11%, *p* < 0.01) after adjustment for BMI, age and sex obtained by log-binomial regression model. This was observed also for patients with sleep apnoea where the accuracy was 66.07% compared to 33.93% in patients without sleep apnoea (*p* = 0.0597 after adjustment for BMI, age and sex).

## Discussion

Results of our study suggest that Mifflin-St Jeor equation has the highest performance for both accuracy and bias, particularly in patients with more severe and complicated obesity. The best accuracy of the Mifflin-St Jeor equation was recorded in patients with type 2 diabetes and sleep apnoea, whereas this formula has a very low accuracy in patients with hypertension who represent the largest percentage of the obese population. It must be emphasized, however, that the accuracy of the Mifflin-St Jeor equation is far from being an ideal tool to correctly predict REE, leading to the conclusion that in obese individuals, it is better to plan a diet intervention by measuring rather than estimating the REE. In this regard, a dietary program set on the REE measured with the indirect calorimetry was shown to be more effective than based on the Harris Benedict equation in promoting weight loss in overweight or obese subjects (20). It would be interesting in the future to verify the efficacy of nutrition plans based on the Mifflin-St Jeor equation. Our results support the use of the Mifflin-St Jeor equation when the indirect calorimeter is unavailable, as recommended by the Academy of Nutrition and Dietetics for obese individuals (8, 9). This equation allows reducing the error brought by using the Harris-Benedict and FAO/WHO/ONU formulas that are still the most used formulas in the clinical practice regardless of the subject's phenotype. In agreement, a recent systematic literature reviews concluded that the Mifflin-St Jeor equation gives the most acceptable REE prediction in obese subjects (21). We observed that the performance of the formulas and in particular of Mifflin-St Jeor equation improves with the increase in the number of comorbidities, which was also associated with the increase in BMI and energy needs. The more likely hypothesis for the good performance of Mifflin St-Jeor equation is that the RENO Diet-Heart cohort of obese individuals in whom this equation was developed, though described as healthy (7), probably had similar, but under evaluated, chronic comorbidities than our cohort.

In conditions with highest energy needs such as obese individuals with ≥3 comorbidities and type 2 diabetes, the Muller BC equation had an accuracy similar to that of Mifflin-St Jeor equation likely because it takes into account the FM, whose inflammation may increase energy requirements.

Though BMI may be the major determinant of energy needs, specific pathologies seem to influence the measured REE at similar levels of BMI. This finding suggests that specific comorbidities may be accompanied by alterations of the organ metabolic rate/functional mass that modify the energy needs in a way not currently predicted by the equations. It is difficult to ascertain how much a greater measured REE contributes to improvement in the accuracy of the predictive equations, because REE, BMI and clinical severity are parameters highly interrelated. Alternatively, as all equations are based on a linear regression model, it is possible that mathematical reasons justify the parallel increase in the accuracy of the formulas and the measured REE. Since even the application of an artificial neural network did not allow to substantially improve the REE prediction in obese subjects (22), it would be more appropriate to change the approach when prescribing a diet, considering also the ability to mobilize the energy stores over the time rather than the energy demand at rest (23).

The strength of our study is that it was conducted in a large sample of unselected obese patients characterized for type and number of comorbidities representing the more frequent condition in which energy requirements are estimated. Several limitations of the study must be underlined. First, the sample was composed by a homogeneous ethnic group of Caucasian origin and whether formulas perform similarly in other ethnic groups should be verified. Second, we did not collect information on the ongoing pharmacological therapy, and thus we cannot distinguish the effect of comorbidities from that of the associated pharmacological therapy. This should be assessed in future studies.

In conclusion, the Mifflin-St Jeor equation, though far from being an ideal tool to precisely predict the REE, should be preferred to other equations to estimate the energy needs of Caucasian morbidly obese patients when the measurement of REE cannot be implemented.

Future studies focusing on the clinical differences that determine the high inter-individual variability of the precision of the REE predictive equations (e.g., on the organ-tissue metabolic rate), could help to develop predictive equations with a better performance.

## Author contributions

RC: data analysis and interpretation, drafting the article, final approval of the version to be published. DS: statistical analysis and data discussion, final approval of the version to be published. AB: data collection and critical revision of data, final approval of the version to be published. MS: data collection, final approval of the version to be published. AT: data collection, final approval of the version to be published. SM: data collection, final approval of the version to be published. PM: data collection, final approval of the version to be published. AZ: statistical analysis supervision, critical revision of the manuscript, final approval of the version to be published. CI: design of the study, supervision and coordination of the work, data analysis and interpretation, wrote the manuscript, critical revision, final approval of the version to be published.

### Conflict of interest statement

The authors declare that the research was conducted in the absence of any commercial or financial relationships that could be construed as a potential conflict of interest.
